# Diagnostic yield of clinical exome sequencing in patients with suspected hereditary neuropathy

**DOI:** 10.55730/1300-0144.6105

**Published:** 2025-10-19

**Authors:** Can Ebru BEKİRCAN-KURT, Doruk ARSLAN, Naz GÜLERAY, Sevim ERDEM-ÖZDAMAR, Burcu BALCI-HAYTA, Ersin TAN

**Affiliations:** 1 Department of Neurology, School of Medicine, Hacettepe University, Ankara, Turkiye; 2 Department of Neurology, Sincan Research and Training Hospital, Ankara, Turkiye; 3 Department of Medical Genetics, School of Medicine, Hacettepe University, Ankara, Turkiye; 4 Department of Medical Biology, School of Medicine, Hacettepe University, Ankara, Turkiye

**Keywords:** Clinical exome sequencing, hereditary neuropathies, diagnostic utility

## Abstract

**Background/aim:**

Hereditary neuropathies exhibit significant genetic heterogeneities, often making molecular diagnosis challenging. Clinical Exome Sequencing (CES) allows the simultaneous evaluation of a wide range of candidate genes, and can be considered an efficient approach to identifying underlying genetic causes. The present study assesses the diagnostic utility of CES in patients with clinically suspected hereditary neuropathy.

**Materials and methods:**

Included in the study were 21 patients with clinically suspected hereditary neuropathy who underwent CES. DNA samples were isolated from peripheral blood and subjected to CES on an Illumina NextSeq 2000 system, while a bioinformatics analysis and variant interpretation were performed using Sophia DDM® software. All identified variants were classified according to the most recent American College of Medical Genetics and Genomics (ACMG) guidelines.

**Results:**

Pathogenic or likely pathogenic variants were identified in seven patients (33.3%), of whom six (28.5%) had a confirmed molecular diagnosis consistent with the clinical phenotype. Variants of uncertain significance (VUS) were detected in nine patients (42.8%), while no clinically relevant variants were found in five (23.8%).

**Conclusion:**

CES contributed significantly to the establishment of molecular diagnoses in nearly one-third of the studied cohort. However, the high prevalence of VUS underscores the limitations of current interpretation frameworks, revealing a need for functional validation and familial segregation analyses. Despite these challenges, CES remains a valuable and appropriate diagnostic tool, particularly in resource-limited healthcare settings.

## 1. Introduction

Hereditary neuropathies, particularly Charcot-Marie-Tooth disease (CMT) and related disorders, constitute a diverse group of peripheral nervous system conditions characterized by progressive motor and sensory dysfunction [1]. Although molecular diagnosis has improved with the identification of common genetic mutations, a substantial proportion of patients remain undiagnosed after standard genetic testing [2].

Clinical Exome Sequencing (CES) has emerged as a powerful tool that facilitates the simultaneous analysis of thousands of genes associated with Mendelian disorders, including both well-established and candidate neuropathy-associated genes. It offers several advantages over targeted gene panels, including an ability to uncover atypical presentations, detect rare or novel mutations, and reanalyze data as new genes are discovered [3, 4]. However, the interpretation of CES findings remains complex. One major limitation is the frequent identification of variants of uncertain significance (VUS), which can complicate clinical decision-making. Furthermore, the clinical utility of CES is often dependent on factors such as detailed phenotyping, variant curation expertise, and the availability of functional and segregation data [3, 4]. The present study evaluates the diagnostic utility of CES in a cohort of patients with clinically suspected hereditary neuropathies, contributing to the growing body of evidence supporting its use in routine diagnostics.

## 2. Materials and methods

### 2.1. Patient selection

Included in the study were patients who presented with clinical and electrophysiological features suggestive of hereditary neuropathy. Baseline evaluations included comprehensive neurological examination, nerve conduction studies, and laboratory tests to exclude acquired causes of neuropathy. Only patients with a suspected hereditary etiology and no evidence of acquired neuropathy were selected for genetic analysis. Typical findings consisted of chronic, slowly progressive distal weakness, muscle atrophy, sensory deficits, foot deformities (e.g., pes cavus), and reduced or absent deep tendon reflexes. Electrophysiological studies revealed axonal or demyelinating polyneuropathy patterns consistent with a hereditary etiology.

### 2.2. CES and Variant Interpretations

Peripheral blood samples were obtained, and CES was performed directly on extracted genomic DNA using the Illumina NextSeq 2000 platform (Illumina, San Diego, CA, USA). Sequencing was performed in the paired-end 150 bp (PE150) format with an average coverage depth of 100x across the targeted regions, and more than 95% bases achieved ≥ 20% coverage. Quality control thresholds were applied uniformly. Reads with coverage depths of <10× or Q-scores of <20 were excluded from the analysis, and only variants with an allele fraction of ≥ 20% were considered. Over 85% of the sequenced bases reached a Q30 quality score, ensuring high-confidence variant calls.

Sequencing data were processed and interpreted using Sophia DDM software (Sophia Genetics, Saint-Sulpice, Switzerland). The analysis pipeline included read alignment (BWA-MEM), variant calling (GATK), Copy Number Variation (CNV) analysis (MuskAT), and mitochondrial variant analysis assessment. A systematic CNV analysis of the *Peripheral Myelin Protein 22* (*PMP22*) gene was conducted as part of the CES evaluation. All CNVs identified in *PMP22* were subsequently confirmed using Multiplex Ligation-dependent Probe Amplification (MLPA) and a SALSA MLPA Probemix P405-A1 kit (MRC-Holland, Amsterdam, The Netherlands).

The identified genetic variants were classified into four categories according to the updated guidelines of the American College of Medical Genetics and Genomics (ACMG) as pathogenic, likely pathogenic, VUS, and no significant findings. Segregation analysis and functional studies were recommended for VUS cases where feasible to clarify variant pathogenicity.

## 3. Results

CES was performed on 21 patients (14 male; 7 female) with a mean age of 37.09 ± 13.73 years. A parental history of consanguinity was noted in eight patients (38.1%). Electrophysiological studies revealed axonal neuropathy in 14 patients (66.6%) and demyelinating features in seven (33.4%). A summary of clinical and genetic findings is presented in Table.

Pathogenic or likely pathogenic variants were identified in seven of the cases (33.3%), two of whom (Cases 3 and 4) were diagnosed with CMT type 1A (CMT1A) based on the detection of heterozygous *PMP22* duplications in the CNV analysis conducted on the Sophia platform, and confirmed subsequently by MLPA. Among the remaining five, four had CES findings consistent with their clinical diagnosis, allowing molecular confirmation. These included:

- A homozygous splice-site variant in the Membrane metalloendopeptidase (*MME*) gene consistent with autosomal recessive CMT type 2T (CMT2T).
- A homozygous deletion-insertion variant in the Mitochondrial inner membrane protein MPV17 (*MPV17*) gene, associated with CMT type 2E (CMT2E).
- A homozygous missense variant in the Histidine triad nucleotide-binding protein 1 (*HINT1*) gene, linked to axonal neuropathy with neuromyotonia.
- A heterozygous variant in the Solute carrier family-5 member-7 (*SLC5A7*) gene, associated with autosomal dominant distal hereditary motor neuropathy type 7 (dHMN7).

A heterozygous pathogenic variant in the Ryanodine receptor 1 (*RYR1*) gene (c.4405C>T, p.(Arg1469Trp)) was detected in one patient (Case 7). Although this variant is classically associated with malignant hyperthermia susceptibility, the clinical phenotype of the patient – including long-standing distal muscle weakness, frequent ankle sprains, high-arched feet, and slowly progressive difficulty with fine motor tasks, as well as chronic sensorimotor axonal changes revealed in electrophysiological studies – did not align with the known *RYR1*-related conditions. This finding was therefore considered likely incidental and unrelated to the patient’s neuropathy. Overall, a confirmed molecular diagnosis was established in six of the 21 patients (28.5%) from the CES analysis.

VUS were identified in nine patients (42.8%). Notably, one patient (Case 11) harbored two distinct heterozygous VUS (c.4958A>G and c.16477C>T) in the Dystonin (*DST*) gene, raising the possibility of compound heterozygosity and indicating a need for segregation analysis. Additional VUS were identified in genes such as Sodium voltage-gated channel alpha subunit 9 (*SCN9A*), Spectrin repeat containing nuclear envelope protein 2 (*SYNE2*), DNA polymerase gamma 2 (*POLG2*), Transient receptor potential vanilloid 4 (*TRPV4*), and Alsin Rho guanine nucleotide exchange factor (*ALS2*). All of these genes have been previously associated with neurogenetic syndromes, but lack definitive genotype-phenotype correlations in these patients. In addition, three patients (Cases 9, 10, and 13) were identified with multiple concurrent VUS in different genes, further complicating interpretation and suggesting potential polygenic or modifier effects.

No clinically significant variants were identified in five patients (23.8%), despite the presence of suggestive clinical and electrophysiological features. These cases highlight the current diagnostic limitations of CES in fully elucidating the genetic etiology of hereditary neuropathies.

## 4. Discussion

This observational study highlights both the utility and limitations of CES in patients with suspected hereditary neuropathy. A molecular diagnosis was achieved in 28.5% of the cases, which is consistent with previous studies reporting diagnostic yields ranging from 20% to 40% in genetically heterogeneous neuromuscular cohorts [5–9]. The identification of pathogenic variants in well-established neuropathy-associated genes such as *PMP22*, *MME*, *MPV17*, and *HINT1* confirms the value of CES, particularly when traditional single-gene screening is inconclusive.

Interestingly, one patient was found to carry a pathogenic variant in *RYR1*, a gene primarily associated with malignant hyperthermia and congenital myopathies. Given the absence of relevant clinical features in this patient, this variant is likely incidental. This case underscores the need to correlate molecular findings with the patient’s phenotype to avoid overinterpretation and potential misdiagnosis.

The detection of VUS in 42.8% of patients reflects a common challenge when interpreting CES results. In one notable case, two heterozygous variants were found in the same gene, *DST*, raising the possibility of compound heterozygosity. While each variant alone might be insufficient to establish pathogenicity, their biallelic presence in trans could be clinically significant [10]. This highlights the importance of segregation analysis and, when feasible, RNA-based or functional studies to clarify the impact of such findings. Furthermore, several patients were identified with multiple VUS in different genes. While the potential cumulative or interactive effect of these variants remains unclear, it may suggest polygenic influences or modifier gene effects – mechanisms that are still poorly understood in the context of peripheral neuropathy [9].

Despite the comprehensive nature of CES, 28.5% of patients had no reportable variants. This may reflect the current limitations of CES in fully elucidating the genetic etiology of hereditary neuropathies, and its failure to identify mutations located in deep intronic or regulatory regions, which are not typically captured by exome-based methods [11, 12]. Additionally, structural variants such as large deletions, duplications, or repeat expansions may escape detection by standard analytical pipelines [11, 13]. Another consideration is that causative variants may reside in genes not yet associated with disease, underscoring the need for gene discovery studies [7]. These findings emphasize the need to integrate CES with complementary technologies, such as long-read sequencing, whole genome sequencing (WGS), or transcriptome analysis by RNA-seq, particularly in unresolved cases [14,15]. Despite the above, CES remains a valuable first-tier diagnostic tool, especially in resource-limited settings where sequential single-gene testing may be impractical.

In conclusion, while CES provides substantial diagnostic value in hereditary neuropathies, its interpretation must be carefully contextualized within the clinical scenario. The high prevalence of VUS and incidental findings highlights the need for a multidisciplinary approach that includes segregation analyses, functional assays, and expert variant reviews. The main limitations of this study include its relatively small sample size and its single-center design, which may restrict the generalizability of the findings. Larger, multi-center studies are necessary to validate and expand upon these findings. CES continues to serve as a key component of the modern diagnostic framework for inherited neuromuscular disorders, although the necessary precautions should be taken.

## Figures and Tables

**Table:** Clinical and genetic findings of individual cases (M: Male, F: Female, ENMG: Electroneuromyography, ACMG: American College of Medical Genetics and Genomics, VUS: Variants of uncertain significance)

Case	Gender	Age	ENMG Findings	ACMG Classification	Gene(s)	Variant Description	Zygosity
**1**	M	42	Axonal	Pathogenic	*MME*	c.957+1G>A	Homozygous
**2**	F	49	Axonal	Pathogenic	*MPV17*	c.263_265delinsTGT	Homozygous
**3**	F	27	Demyelinating	Pathogenic	*PMP22*	Duplication of exons 1–4	Heterozygous
**4**	F	35	Demyelinating	Pathogenic	*PMP22*	Duplication of exons 1–4	Heterozygous
**5**	M	22	Axonal	Likely Pathogenic	*HINT1*	c.283C>G	Homozygous
**6**	M	35	Axonal	Likely Pathogenic	*SLC5A7*	c.769T>C	Heterozygous
**7**	F	48	Demyelinating	Likely Pathogenic	*RYR1*	c.4405C>T	Heterozygous
**8**	M	27	Demyelinating	VUS	*PNKP*	c.991T>G	Heterozygous
**9**	F	65	Axonal	VUS	*DST* *SYNE2 NOP56*	c.5255C>A c.9158G>A c.1561G>C	Heterozygous
**10**	F	46	Axonal	VUS	*DST* *IGHMBP2*	c.16477C>T c.1338G>T	Heterozygous
**11**	F	28	Axonal	VUS	*DST* *DST*	c.4958A>G c.16477C>T	Heterozygous
**12**	M	64	Axonal	VUS	*SCN9A*	c.740A>T	Heterozygous
**13**	M	50	Demyelinating	VUS	*ALS2* *ALS2 SPG11*	c.1815+2_1815+6delinsCAAG c.3797C>G c.6907C>G	Heterozygous
**14**	M	38	Axonal	VUS	*SYNE2*	c.17583G>T	Heterozygous
**15**	M	24	Demyelinating	VUS	*POLG2*	c.1172C>T	Homozygous
**16**	M	37	Axonal	VUS	*TRPV4*	c.796G>T	Heterozygous
**17**	M	53	Demyelinating	None	None	None	None
**18**	M	30	Axonal	None	None	None	None
**19**	M	25	Axonal	None	None	None	None
**20**	M	23	Axonal	None	None	None	None
**21**	M	22	Axonal	None	None	None	None
